# Contribution of NFP LysM Domains to the Recognition of Nod Factors during the *Medicago truncatula/Sinorhizobium meliloti* Symbiosis

**DOI:** 10.1371/journal.pone.0026114

**Published:** 2011-11-08

**Authors:** Sandra Bensmihen, Françoise de Billy, Clare Gough

**Affiliations:** 1 Laboratoire des Interactions Plantes-Microorganismes (LIPM), Institut National de la Recherche Agronomique, Castanet-Tolosan, France; 2 Laboratoire des Interactions Plantes-Microorganismes (LIPM), Centre National de la Recherche Scientifique, Castanet-Tolosan, France; Instituto de Biología Molecular y Celular de Plantas, Spain

## Abstract

The root nodule nitrogen fixing symbiosis between legume plants and soil bacteria called rhizobia is of great agronomical and ecological interest since it provides the plant with fixed atmospheric nitrogen. The establishment of this symbiosis is mediated by the recognition by the host plant of lipo-chitooligosaccharides called Nod Factors (NFs), produced by the rhizobia. This recognition is highly specific, as precise NF structures are required depending on the host plant. Here, we study the importance of different LysM domains of a LysM-Receptor Like Kinase (LysM-RLK) from *Medicago truncatula* called *Nod factor perception* (NFP) in the recognition of different substitutions of NFs produced by its symbiont *Sinorhizobium meliloti*. These substitutions are a sulphate group at the reducing end, which is essential for host specificity, and a specific acyl chain at the non-reducing end, that is critical for the infection process. The NFP extracellular domain (ECD) contains 3 LysM domains that are predicted to bind NFs. By swapping the whole ECD or individual LysM domains of *NFP* for those of its orthologous gene from pea, *SYM10* (a legume plant that interacts with another strain of rhizobium producing NFs with different substitutions), we showed that *NFP* is not directly responsible for specific recognition of the sulphate substitution of *S. meliloti* NFs, but probably interacts with the acyl substitution. Moreover, we have demonstrated the importance of the NFP LysM2 domain for rhizobial infection and we have pinpointed the importance of a single leucine residue of LysM2 in that step of the symbiosis. Together, our data put into new perspective the recognition of NFs in the different steps of symbiosis in *M. truncatula*, emphasising the probable existence of a missing component for early NF recognition and reinforcing the important role of NFP for NF recognition during rhizobial infection.

## Introduction

In plants, many receptor like kinases (RLKs) are involved in developmental responses, as well as in biotic and abiotic stress responses ([1] for review). RLKs are transmembrane proteins whose extracellular domains are responsible for perceiving a specific signal. Analysis of the Arabidopsis genome found, for instance, more than 600 RLKs that group in more than 21 subfamilies, depending on the structural features of their extracellular domains [2]. Among all the possible extracellular domains, Lysin Motif (LysM) domains have recently emerged to be involved in symbiotic and pathogenic interactions [3]. These LysM domains, originally found in bacteria [4], are well represented in plant genomes [5] and some of them were recently shown to bind directly to chitin or chito-oligosaccharides [6]–[9]. What is striking in LysM-RLKs (which are only present in the plant kingdom) is that they usually have 3 LysM domains (LysM1, LysM2, LysM3) in their extracellular region and that each of these LysM domains is quite different in sequence. However, a certain degree of conservation can be observed in the same “ranked” LysM domain among homologous proteins from different plants [5] i.e LysM2 from a rice protein is more similar to LysM2 from a similar *Lotus japonicus* protein than to LysM1 or LysM3 from the same rice protein [10], [11]. Although a few studies have addressed specific properties of plant LysM domains [6], [8], [9], [12], none of them have studied the respective contribution of each LysM domain within the same extracellular domain. In contrast, in cases of other repeated extracellular motifs such as Leucine Rich Repeats (LRRs), it has been shown that motifs are not functionally equivalent ([13], [14] and references herein).

In legumes, putative Nod factor (NF) receptors are LysM-RLKs [10], [11], [15]. Nod factors are lipo-chitooligosaccharides with substitutions at both their reducing and non reducing ends that control the specificity of the legume/rhizobia symbiotic interaction [16]. This interaction leads to the establishment of a root nodule nitrogen fixing symbiosis that is of great ecological and agronomical interest as it enables plants to grow independently of a nitrogen source in the soil. The specificity of the interaction is striking. For instance *Medicago truncatula* can only be nodulated by *Sinorhizobium meliloti* bv. *meliloti* (*S. meliloti*) and *S. medicae*. *S. meliloti* produces mainly tetrameric NFs (i.e backbone of four N-acetyl Glucosamine (GlcNAc) residues) with a sulphate substitution at the reducing end, and an O-acetate and a C16:2 lipid chain substitutions at the non reducing end [16]. These structural features are critical, as *S. meliloti* mutants that produce NFs with modified substitutions are no longer properly recognised by *M. truncatula*, with the sulphate substitution being more critical for symbiotic responses than the non reducing end substitutions [17]–[19].

In *M. truncatula*, the *NFP* (*Nod Factor Perception*) gene encodes a LysM-RLK with an inactive kinase domain [11]. *NFP* is the gene in which mutations result in the earliest block in the *M. truncatula/S. meliloti* interaction. Indeed, *nfp* mutants do not show any response following NF treatment and are completely deficient for nodulation [20]. LYK3 is another symbiotic LysM-RLK from *M. truncatula* that, unlike NFP, displays an active kinase activity [11], [21], [22]. Also, unlike *nfp* mutants, *lyk3* mutants are not impaired in the very early responses to NFs (such as calcium spiking or early gene expression), but are blocked for the infection and nodulation processes [23]. Thus, in contrast to *Lotus japonicus* where two LysM-RLKs were shown to be required for early symbiotic events [10], [15], in *M. truncatula*, only NFP was so far shown to be necessary for early NF responses. Whether NFP is the only gene required to mediate these early symbiotic steps remains an open question as no genetic approach has identified another *M. truncatula* mutant phenotype resembling that of *nfp* mutants. Moreover, although homology modelling and NF docking studies predict that LysM domains of NFP bind NF [24], such binding has not yet been reported for any putative NF receptor. While data indicate that LYK3 is involved in the recognition of specific NF structures [25], nothing of this sort is known for NFP and genetic analysis remains a major tool to test the function of this protein in NF recognition.

In order to test the importance of the LysM domains in the extracellular domain (ECD) of NFP in the recognition of *S. meliloti* NFs, we tested the ability of a chimeric construct bearing the ECD of SYM10, the orthologous protein from pea (*Pisum sativum*), under the *NFP* promoter (pNFP:SYM10-NFP construct), to complement *nfp* mutant roots. Pea belongs to a different cross-inoculation group and its symbiont, *Rhizobium leguminosarum* bv. *viciae*, which is not able to nodulate with *M. truncatula*, produces non sulphated NFs, with a different type of fatty acid on its non-reducing end compared to *S. meliloti* NFs [26]. Pea and *M. truncatula* belong to the same clade [27] and the *sym10* and *nfp* mutants display very similar phenotypes [10], [11]. Moreover, SYM10 and NFP share 87% protein sequence similarity [11] and we took advantage of this conservation to search for regions involved in specific NF recognition. In the *M. truncatula/S. meliloti* interaction, we know that *NFP* controls both early responses (leading for instance to the expression of the nodulin gene *MtENOD11*), and entry of rhizobia into the plant [11]. Therefore, we used as biological tests for complementation both *pMtENOD11:GUS* induction [28] and rhizobial infection and nodule development. As infection and nodule organogenesis were not restored with the pNFP:SYM10-NFP construct upon *S. meliloti* inoculation, we took advantage of this difference to analyse individual LysM swaps between NFP and SYM10. Our results revealed a functional specialisation among the three different LysM domains of NFP, with LysM2- and one of its leucine residues in particular- being critical for infection. In conclusion, we provide new insights on the role of NFP for the recognition of NF substitutions at different steps of symbiosis.

## Results

### Testing *NFP* chimeric constructs for *nfp* complementation

**Figure 1:**
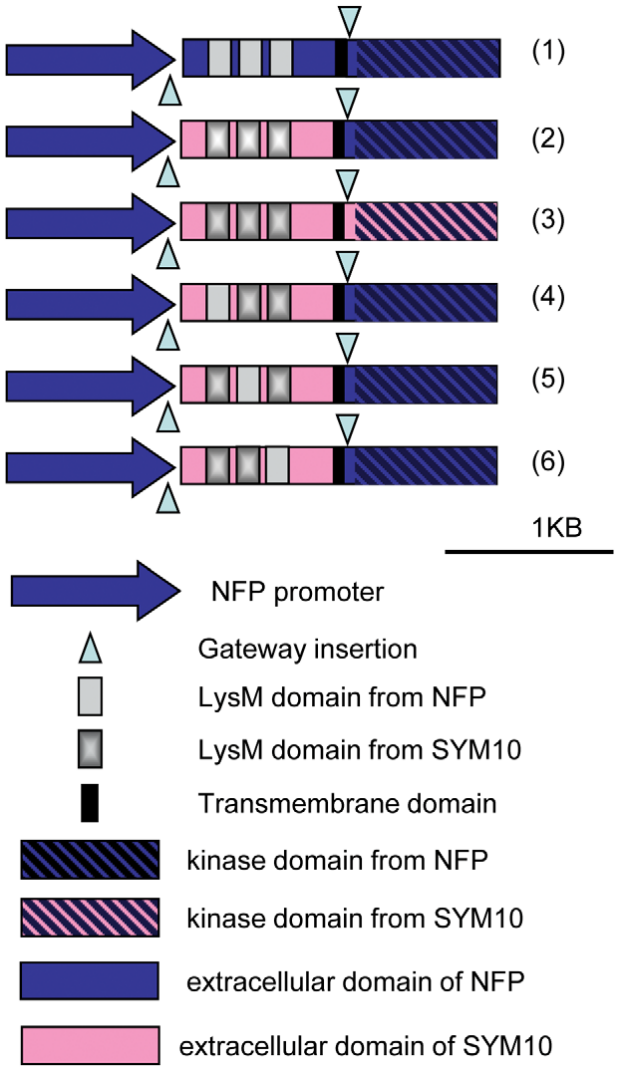
Schematic representation of the Gateway® constructs used. Schematic drawing of the genes obtained after “Multisite Gateway® ” LR recombination. pNFP:NFP-NFP (1), pNFP:SYM10-NFP (2), pNFP:SYM10-SYM10 (3), LysM1 swap (4), LysM2 swap (5), LysM3 swap (6). The Gateway® recombination reaction leaves a 27 nt supplementary sequence that is indicated by a triangle.

To study the role of the NFP ECD in recognition of sulphated NFs, we generated chimeric constructs with the ECD of either SYM10 or LYK3 (up to the beginning of the transmembrane domain) and the juxtamembrane and kinase part of NFP, under the control of the *NFP* promoter (*i.e* pNFP:SYM10-NFP and pNFP:LYK3-NFP). In addition to these constructs made by classical cloning methods, we also developed a more flexible way to swap the ECD of NFP, using the Multisite Gateway® technology, and used it to make further chimeric constructs (Fig. 1).

The Multisite Gateway® technology enables reconstruction of three fragments (namely promoter, ECD and kinase domain in our case) by recombination and we placed recombination sites at the end of the promoter and after the transmembrane domain. As this recombination leaves a 27 bp sequence in between the different fragments, we first compared “classical” constructs and Gateway® constructs for the ability of pNFP:NFP-NFP to complement *nfp* mutants for nodulation and found comparable complementation abilities (64% versus 63% of plants nodulated at 21 days post inoculation (dpi)). We also compared both types of constructs for restoration of *pMtENOD11:GUS* expression in *nfp* roots at 7 dpi (Table S1). As there was no significant difference between classical and Gateway® constructs, we used both of them for whole ECD analysis but used the flexibility of the Gateway® system for subsequent domain swaps and site-directed mutagenesis.

### The ECD of SYM10 from pea does not change the *Medicago truncatula* requirement for sulphated NFs

**Figure 2:**
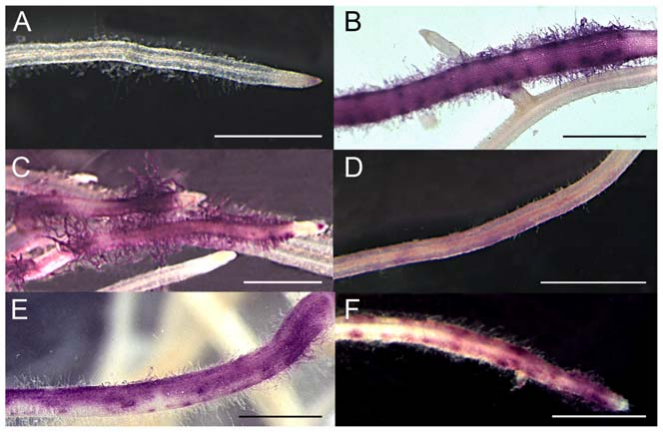
The extracellular domain of SYM10 can replace that of NFP for activation of *pMtENOD11:GUS*. *nfp pMtENOD11:GUS* roots untransformed (A) or transformed by pNFP:NFP-NFP (B), pNFP:SYM10-NFP (C), pNFP:LYK3-NFP (D) or pNFP:SYM10-SYM10 (E) and stained for GUS activity 7 dpi with wild type *S. meliloti*. *nfp lyk3 pMtENOD11:GUS* roots transformed by pNFP:SYM10-NFP (F). GUS activity is shown in magenta. Bars = 1 mm.

We took advantage of a *nfp pMtENOD11:GUS* line [11] to test the ability of both pNFP:LYK3-NFP and pNFP:SYM10-NFP to complement for *pMtENOD11:GUS* expression, 7 dpi with *S. meliloti* wild type strain. As previously described [11], control *nfp pMtENOD11:GUS* plants did not show any symbiotic GUS expression upon rhizobium inoculation (Fig. 2A, note that the GUS staining in the root tip is from constitutive expression of *pMtENOD11:GUS* [28]) and, as expected, most pNFP:NFP-NFP transformed *nfp pMtENOD11:GUS* plants showed strong symbiotic *pMtENOD11:GUS* expression (Fig. 2B, Table 1). Surprisingly, the pNFP:SYM10-NFP constructs (cloned either by classical or Gateway® cloning systems) could also restore strong symbiotic *pMtENOD11:GUS* expression and in a similar proportion of transformed plants compared to pNFP:NFP-NFP (Fig. 2C, Table 1, Table S1). In contrast, the pNFP:LYK3-NFP construct only gave weak *pMtENOD11:GUS* expression (Fig. 2D) and in a significantly lower number of plants (Table 1).

**Table 1:** Number of plants showing *pMtENOD11:GUS* induction in *nfp pMtENOD11:GUS* roots transformed with pNFP:NFP-NFP, pNFP:SYM10-NFP or pNFP:LYK3-NFP, 7 dpi with *S. meliloti* wild type or *S. meliloti nodH*.

	pNFP:NFP-NFP	pNFP:SYM10-NFP	pNFP:LYK3-NFP	pNFP:SYM10-SYM10
*S. meliloti* wild type	30/38^a^ (79%)	33/43^a^ (77%)	13/43 (30%)	18/35 (51%)
*S. meliloti nodH*	7/15^b^ (47%)	12/31^b^ (39%)	NT	NT

The same superscript letter indicates that these proportions are not statistically different, as shown by a chi-square test of independence.

NT: Not Tested.

The total number of plants tested comes from 3 to 9 independent experiments.

This ability of the pNFP:SYM10-NFP construct to confer symbiotic *pMtENOD11:GUS* expression was not dependent on the NFP kinase domain, as strong GUS expression was observed in *nfp* plants carrying the pNFP:SYM10-SYM10 construct, where the ECD of SYM10 is fused to the kinase part of SYM10 (Fig. 1, Fig. 2E, Table 1). Also, *LYK3* was not necessary for the GUS activity conferred by the pNFP:SYM10-NFP construct as *pMtENOD11:GUS* induction was still seen after introduction of the pNFP:SYM10-NFP construct into a *nfp lyk3* mutant (Fig. 2F). To confirm that the observed *MtENOD11* induction was due to NF recognition, we treated pNFP:SYM10-NFP-transformed *nfp pMtENOD11:GUS* plants with wild-type (sulphated) *S. meliloti* NFs and observed the same ability to induce *pMtENOD11:GUS* expression as in wild type plants (Fig. S1, compare C and B).

We conclude that, in *M. truncatula*, the SYM10 ECD is as efficient as that of NFP to activate the signalling pathway for *MtENOD11* induction following perception of *S. meliloti* NFs. We also conclude that a “NFP like” ECD is needed as LYK3 is much less efficient (the NFP ECD shows 77% and 21% protein sequence identity to the ECDs of SYM10 and LYK3, respectively).

**Figure 3:**
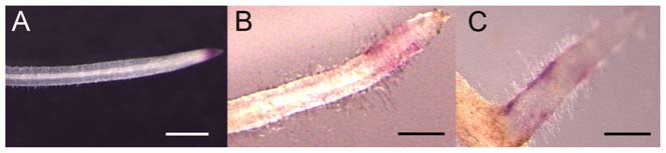
The extracellular part of SYM10 does not increase the recognition of non sulphated NF. *nfp pMtENOD11:GUS* roots untransformed (A) or transformed with pNFP:NFP-NFP (B), or pNFP:SYM10-NFP (C), 7 dpi with *S. meliloti nodH*. GUS activity is shown in magenta. Bars = 500 µm.

Since the symbiont of pea, *R. leguminosarum* bv. *viciae*, produces non sulphated NFs, we tested the ability of *nfp pMtENOD11:GUS* plants transformed by pNFP:SYM10-NFP to respond to non sulphated NFs. These NFs were obtained from the *S. meliloti* mutant *nodH* [17](Fig. 3, Fig. S1)) or purified from *R. leguminosarum* bv. *viciae* (Fig. S1). As expected, only very weak and restricted *pMtENOD11:GUS* expression was induced in pNFP:NFP-NFP *nfp* transformed plants after *S. meliloti nodH* inoculation (Fig. 3B). A similar weak *MtENOD11* expression was seen with pNFP:SYM10-NFP (compare Fig. 3C to Fig. 3B, Fig. S1F to Fig. S1E), even with NFs produced by *R. leguminosarum* bv. *viciae* (Fig. S1I and S1H). The frequency of GUS responding plants to *S. meliloti nodH* was also similar between pNFP:NFP-NFP and pNFP:SYM10-NFP *nfp* transformed plants (Table 1). Moreover, no nodules were ever seen when pNFP:SYM10-NFP *nfp* transformed roots were inoculated with *S. meliloti nodH*, even at 21 dpi. These results are in accordance with the SYM10 and NFP ECDs being equivalent, in a *M. truncatula* background, for early recognition of *S. meliloti* NFs.

### The three LysM domains of NFP do not have equivalent roles and LysM2 is crucial for infection

**Figure 4:**
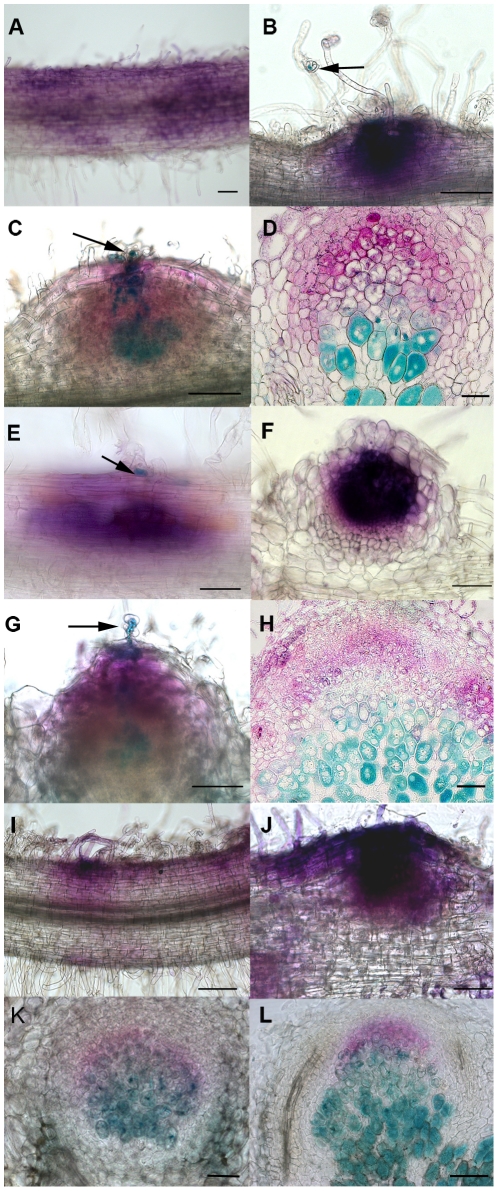
LysM swap constructs reveal different NFP LysM domain requirements for infection. *nfp pMtENOD11:GUS* plants were transformed with pNFP:SYM10-NFP (A, B), pNFP:NFP-NFP (C, D), LysM1 swap (E, F), LysM2 swap (G, H), LysM3 swap (I, J) or pNFP:SYM10-P154L-NFP (K, L). *pMtENOD11:GUS* activity (magenta) and rhizobial infection (revealed by lacZ activity, in blue) are shown in roots and nodule sections at 7 dpi (A, C, E, G, I, K) and 21 dpi (B, D, F, H, J, L) with wild type *S. meliloti*. Micro-colonies in curled root hairs are indicated by arrows in B, C, E, G. Bars = 100 µm. D and H are microtome 5 µm-thick sections and F, J, K, L 70 µm-thick vibratome sections.

The ability of the pNFP:SYM10-NFP construct to complement *nfp* roots inoculated with wild type *S. meliloti* for nodulation was clearly different compared to pNFP:NFP-NFP. Indeed, no cell division or infection thread (IT) formation could be seen with the pNFP:SYM10-NFP construct at 7 dpi (Fig. 4A) (43 plants, n = 9), and no nodules were seen, whereas at this stage, most of the pNFP:NFP-NFP plants had started to nodulate (Fig. 4C). So, the SYM10 ECD was much less efficient than that of NFP for infection and nodulation and we exploited this difference to dissect the importance of individual LysM domains of NFP for these symbiotic processes.

**Figure 5:**
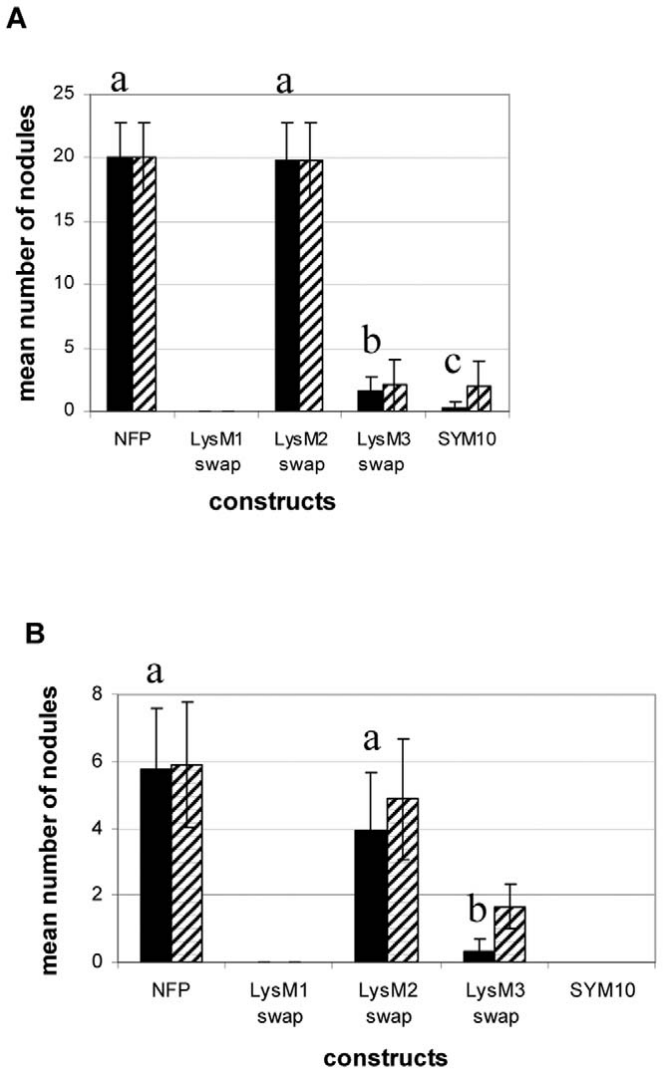
LysM domains of NFP do not have equivalent functions for nodulation. Nodules were counted on *nfp* roots transformed with pNFP:NFP-NFP (“NFP”), LysM1 swap, LysM2 swap, LysM3 swap and pNFP:SYM10-NFP (“SYM10”) constructs, 21 dpi with wild type *S. meliloti* in a sepiolite/sand mixture (A) (nodulation was assayed for 12 to 37 plants) or in growth pouches (B) (nodulation was assayed for 11 to 32 plants). Bars correspond to the 95% confidence interval. Black bars represent average nodule numbers on the total number of plants tested, and hatched bars represent mean numbers of nodules on nodulated plants only. In each condition, a Mann-Whitney statistical test was performed on the number of nodules observed on the total number of plants and the superscript letter corresponds to the different categories obtained in that way (i.e the same letter indicates a similar mean, within the same panel).

We made three different SYM10 ECD variants, differing by which LysM domain of *SYM10* was replaced by that from *NFP*, and named these constructs LysM1 swap, LysM2 swap and LysM3 swap (Fig. 1). We normally used a “growth pouche” system, which enables the kinetics of nodulation to be followed but in the case of the individual LysM swaps, two experimental conditions were assayed. First, a “high-nodulating” medium (sand/sepiolite mixture) was used to enhance nodulation efficiency (see Fig. 5A), then the “growth pouche” system (Fig. 5B) was also tested.

In both conditions, pNFP:NFP-NFP *nfp* plants inoculated with wild type *S. meliloti* started nodulating at 7 dpi (Fig. 4C) and displayed mature nodules at 21 dpi (Fig. 4D). With the pNFP:SYM10-NFP construct, we could only see occasional bumps and abnormal abortive ITs at the late time point of 21 dpi (Fig. 4B, arrow), although, in one experiment where plants were grown in the sand/sepiolite medium, 2/14 plants did nodulate (2 nodules/plant, versus an average of 20/plant for pNFP:NFP-NFP constructs (Fig. 5A)). For the individual LysM swaps introduced in *nfp* mutant plants, LysM1 could restore cell divisions and showed rhizobia entrapped in root hair curls but no IT formation at 7 dpi (Fig. 4E, arrow) and non infected bumps at 21 dpi (Fig. 4F). No nodules were observed (Fig. 5A and 5B). In contrast, changing the LysM2 domain of SYM10 for that of NFP was enough to restore normal infection, as early as 7 dpi (Fig. 4G) with comparable number and structure of nodules at 21 dpi as for pNFP:NFP-NFP (Fig. 4H and Fig. 5A and 5B). Finally, replacing LysM3 did not produce either noticeable cell division or infection at 7 dpi (Fig. 4I) and produced large uninfected bumps (Fig. 4J) and a few nodules at 21 dpi (an average of 2 nodules/plant, 9/12 plants in sand/sepiolite medium, Fig. 5A, but only 3/14 plants in pouches, Fig. 5B). This construct was therefore slightly more efficient for nodulation than the original pNFP:SYM10-NFP construct (Fig. 5A and 5B), but was less efficient than the LysM2 swap at restoring nodulation in *nfp* plants.

These data indicate that the LysM2 swap is as efficient as the whole NFP ECD for recognition of *S. meliloti* NFs leading to infection and nodulation.

### Leucine 154 of NFP LysM2 is essential for nodulation

To further understand the importance of LysM2, we searched for a critical residue in LysM2 that might account for efficient NF recognition during infection. Sixteen amino acids (aa) differ between the LysM2 swap and the original pNFP:SYM10-NFP construct, among which 13 are within the LysM2 domain itself and 9 of these belong to different aa classes (Fig. S2A). We first looked at aa predicted to interact with NF by *in silico* docking studies [24]. We also used the alignment of the sequence of LysM2 from five other *Medicago* spp. and from two spp. of the same cross-inoculation group as pea (*Vicia sativa* and *Vicia hirsuta*) and focused on the aa that are conserved in *Medicago ssp.* but differed with pea, *Vicia sativa* and *Vicia hirsuta* (Fig. S2B). In that way, there were only 2 aa that were predicted to be part of the NF binding site, as proposed by Mulder and co-workers, and that had different biochemical properties between the NFP and SYM10 proteins. These were K141 of NFP, predicted to interact with the sulphate group of *S. meliloti* NFs, and L154 of NFP, predicted to interact with the fatty acid chain. We also tested a nearby residue that is different between the *Medicago* and pea and *Vicia* ssp., T156 (Fig. S2B, dotted arrow). Starting from pNFP:SYM10-NFP, we made constructs keeping the whole extracellular part of SYM10 but bearing either the E141K (named pNFP:SYM10-E141K-NFP), P154L (pNFP:SYM10-P154L-NFP) or I156T (pNFP:SYM10-I156T-NFP) mutation. At 7 dpi with wild type *S. meliloti*, *nfp pMtENOD11:GUS* plants transformed with these different point mutation constructs all showed strong *pMtENOD11:GUS* induction (Fig. S3). No nodules were seen on pNFP: SYM10-I156T-NFP (out of 22 plants, Table 2, Fig. S3C), and only one young nodule formed on 1/21 plants with the pNFP:SYM10-E141K-NFP construct, whereas 27/50 plants showed young nodules with pNFP:SYM10-P154L-NFP (Table 2, Fig. 4K, Fig. S3D), compared to 7/10 nodulated plants with the full NFP ECD (Table 2, Fig. S3B). At 21 dpi, a large number of *nfp* plants complemented with the pNFP:SYM10-E141K-NFP and pNFP: SYM10-P154L-NFP constructs, but very few of those bearing the pNFP: SYM10-I156T-NFP construct, showed nodule formation (Table 2). Interestingly, plants transformed with pNFP: SYM10-E141K-NFP or pNFP:SYM10-P154L-NFP construct showed a mean number of nodules that was not statistically different from that obtained with the NFP ECD itself (Table 2). Further analysis showed that nodules formed with the pNFP:SYM10-E141K-NFP or pNFP:SYM10-P154L-NFP construct had normal structures and were normally infected by rhizobia (Fig. 4L and Fig. S4B and S4C). Because of the earlier appearance of nodules (at 7 dpi), it seemed that the P154L mutation was more efficient than E141K to confer to the SYM10 ECD the ability to complement for nodulation. In contrast, the I156T mutation could not confer any nodulation capacities to the SYM10 ECD at 7 dpi or 21 dpi (Table 2, Fig. S3C and Fig. S4D).

**Table 2:** Comparison of nodulation levels of *nfp* plants complemented by constructs with different point mutations either in the SYM10 ECD or NFP ECD, upon inoculation with wild type *S. meliloti*, at 7 dpi or 21 dpi.

Construct	Number of nodulated plants	Mean number of nodules/plant
pNFP:NFP-NFP 7 dpi	7/10	3.3^a,b^
pNFP:SYM10-NFP 7 dpi	0/22	0
pNFP:SYM10-E141K-NFP 7 dpi	1/25	0.04
pNFP:SYM10-I156T-NFP 7 dpi	0/22	0
pNFP:SYM10-P154L-NFP 7 dpi	27/50	1.62^a^
pNFP:NFP-K141E-NFP 7 dpi	7/10	2.64^b^
pNFP:NFP-T156I-NFP 7 dpi	15/26	2.19^b^
pNFP:NFP-L154P-NFP 7 dpi	0/25	0
pNFP:NFP-NFP 21 dpi	27/35	3.26^c^
pNFP:SYM10-NFP 21 dpi	0/19	0
pNFP:SYM10-E141K-NFP 21 dpi	13/16	2.6^c^
pNFP:NFP-K141E-NFP 21 dpi	23/40	2.42^c^
pNFP:NFP-NFP 21 dpi	17/20	5.75^d,e^
pNFP:SYM10-P154L-NFP 21 dpi	41/42	3.90^d^
pNFP:SYM10-I156T-NFP 21 dpi	2/23	0.09
pNFP:NFP-L154P-NFP 21 dpi	0/17	0
pNFP:NFP-T156I-NFP 21 dpi	24/26	6.73^e^

The three groups of results correspond to three series of experiments.

For each construct and time point, two independent experiments were performed.

The means bearing the same superscript are not significantly different following a Kruskal-Wallis (for 7 dpi data) or ANOVA (for 21 dpi data) statistical test.

To confirm the importance of these residues for the ability to nodulate, we made the converse experiment of mutating these residues, one by one in the NFP ECD and tested the ability of these constructs, named “pNFP:NFP-K141E-NFP”, “pNFP:NFP-L154P-NFP” and “pNFP: NFP-T156I-NFP” to complement *nfp* plants for nodulation. For all these constructs, we assessed the nodulation efficiency in pouches after inoculation with *S. meliloti* (Table 2). Interestingly, the K141E mutation did not prevent the ability of this construct to complement the *nfp* mutant for nodulation; the mean number of nodules obtained at 7 dpi and 21 dpi was not statistically different from the values obtained for the unmutated NFP construct (Table 2). In a similar way, the T156I mutation did not seem to prevent the ability to nodulate (Table 2), even at 7 dpi (Table 2, Fig. S3F). In both these cases, the structure and bacterial colonization of the nodules obtained at 21 dpi were similar to those obtained with the wild-type construct (Fig. S4E). However, the pNFP: NFP-L154P-NFP construct did not complement the *nfp* mutant for nodulation, either at 7 dpi or 21 dpi (Table 2 and Fig. S3G and S4F).

Therefore, these results show that replacing P154 in SYM10 by a leucine residue is sufficient to confer nodulation ability to the SYM10 ECD and that L154 is absolutely necessary in NFP for nodulation. In contrast, the K141 residue of NFP could enhance the ability of SYM10 ECD to complement the *nfp* mutation for nodulation, but is not absolutely required for nodulation by the NFP ECD.

## Discussion

### NFP is not directly responsible for recognition of the sulphate substitution of *S. meliloti* NFs for the early steps of NF signalling

To test the implication of the NFP ECD in specific recognition of *S. meliloti* sulphated NFs, we swapped it for the ECD of SYM10, which is the ortholog of NFP from pea, a legume that interacts with rhizobia producing non sulphated NF. We have shown that the SYM10 ECD, but not the LYK3 ECD, can replace that of NFP to trigger early NF signalling in response to sulphated NFs in *M. truncatula*. Indeed, replacing the NFP ECD by that of SYM10 did not change *M. truncatula*'s requirement for sulphated NF structures for this step. This suggests that there is no specific difference between the NFP and SYM10 ECDs that could account for sulphate recognition in *M. truncatula* for early symbiotic gene expression and that the NFP ECD is probably not involved directly in sulphate recognition for early NF signalling events. This is in accordance with non sulphated NF being able to trigger early responses in *M. truncatula*, although at higher concentrations [29], [30], in a *NFP* dependent manner (this study). Moreover, it has been shown recently that the symbiotic arbuscular mycorhizal fungus *Glomus intraradices* produces lipo-chitooligosaccharides (called Myc-LCOs) very similar to NFs and that *NFP* is required for perception of low concentrations of non sulphated Myc-LCOs [31], suggesting that *NFP* mediates both sulphated and non sulphated LCO-induced responses. It was already suggested by Staehelin and coworkers [32] that a putative Medicago NF receptor might be flexible enough to interact with both sulphated and non-sulphated NFs and that the difference in bioactivity could be partly determined by the fact that sulphated NFs are more resistant than non-sulphated ones to degradation by plant chitinases. Taken together, these data suggest that another component is responsible for the ability of *M. truncatula* to respond to sulphated NFs by induction of early nodulin gene expression. This component is not LYK3, as the *pMtENOD11:GUS* activation was not dependent on LYK3 (consistent with *LYK3* being dispensable for early NF responses in *M. truncatula* [23]), but might be another LysM domain protein.

### The NFP LysM2 domain and its Leu 154 residue are critical for infection and nodulation

The SYM10 ECD is, in contrast, poorly able to complement *nfp* mutants for infection and nodulation and, by sequentially replacing one LysM domain of the SYM10 ECD for its equivalent from NFP, we have demonstrated that the three LysM domains of NFP are equivalent to those of SYM10 for early NF signalling but have different functions during the later stages of symbiosis. Thus, while all the LysM swaps could efficiently restore symbiotic *pMtENOD11:GUS* expression in a *nfp* mutant, only the LysM2 swap could restore efficient infection and nodulation. Both a lysine (K141) and a leucine (L154) residue in LysM2 could, individually, enhance the efficiency of SYM10 ECD for nodulation but only the L154 residue was sufficient to restore full nodulation as early as 7 dpi and was essential for complementation by the NFP ECD. A closeby threonine residue (T156) did not improve the capacities of the SYM10 ECD and, when mutated, did not impair the functionality of the NFP ECD. This is consistent with data from *Lotus japonicus* showing that LysM2 is a zone of nucleotide divergence, susceptible to have evolved special roles for nodulation, but where the conserved residues are not all involved in specific NF recognition [33], [34]. Indeed, a single residue from LysM2 of the *L. japonicus* NFR5 protein was responsible for the specific recognition of the non reducing end of *Mesorhizobium loti* NFs [12]. *In silico* binding studies performed by Mulder et al. [24] identified the L154 residue of NFP LysM2 as a possible interactor with the acyl chain of the NF. This, together with our data, indicates that NFP is also involved, as shown for NFR5 in *L. japonicus* and LYK3 in *M. truncatula* [12], [25], in the recognition of substitutions at the non reducing end of NFs. This strengthens previous data indicating that recognition of the non-reducing end of NFs is more important for infection than for early signalling. Indeed, the *nodFL* mutant of *S. meliloti*, that produces NFs with a modified acyl chain and lacking the O-acetate residue at the non reducing end [19] is able to trigger normal early signalling responses leading to the induction of *MtENOD11* but is blocked for infection [19], [21], [29], [35]. L154 is predicted to be part of the β2 strand of NFP LysM2 and is a hydrophobic residue. The change of this residue for a proline in SYM10 is likely to induce a change in the β sheet structure and thus modify the interaction with the acyl chain. This is also consistent with *R. leguminosarum* bv. *viciae* (the pea symbiont) and *S. meliloti* producing different types of acyl chain (see [36] for review). The ability of the K141 residue to partially restore nodulation efficiency in the SYM10 ECD but the fact that it is not absolutely necessary for the NFP ECD function suggests that this residue is involved in the NF binding site but is not essential. The lack of effect observed with the T156I (or I156T in SYM10) mutation is consistent with the absence of any predicted implication of this residue in the NF binding site. Overall, our genetic studies support the model suggested by Mulder and co-workers [24]. Structure-function studies by biochemical approaches (*i.e* binding tests with purified proteins or domains), using various NF structures and NFP variants, would be needed to fully validate the importance of aa involved in the specific recognition of NF.

### Different roles can be assigned to LysM1 and LysM3

By replacing the LysM1 domain of SYM10 by that of NFP we enhanced the capacity of the SYM10 ECD to induce cortical cell divisions (CCD) at an early time point (7 dpi), but infection was blocked at the step of infection thread formation, and no nodules were formed at 21 dpi. Such a phenotype is also characteristic of certain *S. meliloti* mutants producing modified NFs [19], indicating that CCD has less “stringent” NF structural requirements than the infection process. This suggests that the LysM1 domain of NFP confers recognition of NF structural features sufficient for CCD and root hair curling. When we replaced LysM3 of SYM10 by that of NFP, we could improve nodulation of the SYM10 construct (as infected nodules could form at 21 dpi), but this process was delayed and less efficient than with the LysM2 swap. This result suggests that LysM3 also plays a role in infection, although a less important role than that of LysM2. This is consistent with sequence analysis in *L. japonicus* where LysM3 has also been shown to be a site for positive selection [33]. Taken together, our data suggest that all three NFP LysM domains contribute to the function of NFP, but that they may not all interact with the ligand in the same manner, and, as such, have specialised roles. Each LysM domain of NFP may therefore interact with one NF molecule, maybe with different affinities or in a cooperative manner. This is consistent with NMR studies on the interaction of a tandem LysM containing protein from fern with chitin, which suggest that one LysM domain interacts with one chitopentaose fragment [7], and, recently, a binding assay on a fungal protein containing three LysM domains has shown that one LysM domain binds to one oligosaccharide consisting of 5 or 6 GlcNAc residues [37].

Phylogeny analysis of LysM-RLK proteins has shown a diversification event that predated the divergence of monocot and dicot plants [5], [11]. This has led to the three different LysM domains of one protein being more similar to the equivalent domains of other proteins than they are to each other, with LysM2 domains often appearing as the most conserved, even between legume and non legume proteins (Clare Gough, unpublished). This, together with our data, suggests that LysM2 domains fulfil some specific structural requirement within ECDs that are made up of three LysM domains, and, at the same time, contain area(s) of aa variation that are crucial for specific ligand recognition. LysM2 domains of LysM-RLK proteins are therefore candidates of choice to look for specific functional-related features in other types of plant/micro-organism interactions.

### A model for NFP function at the different stages of symbiosis

A model was suggested some years ago, based on both *M. truncatula* and *S. meliloti* mutant phenotypes [19], [38] that postulates two types of NF receptor complexes, one controlling early signalling processes (the “signalling receptor”) and one controlling infection (the “entry receptor”). Primarily, NFP was considered as a signalling receptor and LYK3 as an entry receptor, however, knock down of *NFP* by RNA interference indicated that NFP was also involved in infection [11], and thus part of the entry receptor. Our data reinforce the importance of NFP in both NF perception mechanisms, but with different roles. As non sulphated NFs are at least 10,000 times less active than sulphated ones (and 100 less active that NFs modified on their non reducing end) in early *NFP* dependent responses such as calcium spiking and *MtENOD11* activation [29], [30], [39], it appeared that NFP would control NF structural recognition in the signalling step with a special sensitivity to sulphated NFs. The fact that the NFP and SYM10 ECDs are interchangeable for *MtENOD11* induction upon sulphated NF stimulation suggests that NFP is poorly involved in specific recognition of that substitution during the early steps of NF signalling. Instead, NFP could be responsible (within the signalling receptor) for recognition of the lipo-chitooligosaccharide backbone. LYK3, has been shown to be critical for the recognition of the non reducing end of NF structure necessary for proper infection [23], [25]. Our data show that the NFP LysM2 domain, and L154 in particular, are crucial for the “entry step” of rhizobia, suggesting that NFP, as well as LYK3, is involved in specific NF recognition for infection. Furthermore, our data, together with *in silico* docking studies [24], predict that, like LYK3, this specific recognition of NF would rather be at the non-reducing end of the NF.

Thus, our work modifies our vision of the roles of NFP in symbiosis, emphasising its importance to control infection and supporting the hypothesis of a missing component that would act together with NFP in the early signalling complex to confer the sensitivity to sulphated NF, hence revealing enhanced complexity in the mechanism of NF perception in *M. truncatula*.

## Materials and Methods

### Plant growth conditions

The first nodulation complementation assays by pNFP:NFP-NFP were performed with both *nfp-1* and *nfp-2* mutants. *nfp-2 pMtENOD11:GUS* lines [11] in the cv. Jemalong A17 wild-type background were used for all subsequent complementation experiments. The double mutant *nfp lyk3* was obtained by crossing *nfp-1 pMtENOD11:GUS* to *B56* (*lyk3-1*) *pMtENOD11:GUS* and genotyping F3 offspring (see below).

Surface sterilized seeds were sown on agar plates and placed for 3 days in the dark at 4°C, then left overnight at 25°C to germinate.

For root transformation, we used ARqua1 *Agrobacterium rhizogenes* as described by Boisson-Dernier [40]. *A. rhizogenes* transformed roots were selected by *GFP* expression carried by the pCAMBIA2202 binary vector and by kanamycin resistance. The majority of the selected roots were both kanamycin resistant and GFP+ but long, kanamycin resistant, GFP minus roots were also selected for nodulation assays, as previous studies showed that such roots can form nodules when the pNFP:NFP-NFP construct is used.

For nodulation assays, transformed plants were transferred either to pouches [41] or to sepiolite (Agrauxine, Quimper)/sand (2∶1 volume mix) in pots and grown in a chamber at 25°C with 18 h light/6 h dark cycles [42].

### Rhizobial strains and inoculation

Wild-type *S. meliloti* RCR2011 (pXLGD4) (reference GMI6526) and *S. meliloti* RCR2011 *nodH* (pXLGD4) (reference GMI6527) were grown at 28°C on tryptone yeast medium supplemented with 6 mM calcium chloride and 10 µg/mL tetracycline or 100 µg/mL neomycine and 10 µg/mL tetracycline, respectively.

The bacteria were “scratched” from the plate after 2 days and resuspended in sterile water. The inoculum obtained in that way was adjusted to OD_600_ = 0.2, in water. Plants were inoculated with 2 ml of that inoculum.

### Plasmid constructs

For all cloning experiments, the templates used were p2201-NFP [11] for *NFP*, a *LYK3* cDNA provided by B. Lefebvre (LIPM) and a subcloned *SYM10* DNA fragment of pea cv. Frisson in pGEM®-T vector (pGEMT-SYM10) provided by C. Rosenberg, (LIPM).

#### Classical “restriction site” cloning

To generate the pNFP:NFP-NFP plasmid, we first introduced 1.1 kb from the *NFP* promoter that was amplified using the primers pNFPfor: 5′-ACTCTAGAGGATCCCCATC-3′ and pNFPrev: 5′-TTTCTAGATTGTGAGGAAATGCAAA-3′ (using TaKaRa LaTaq™, Takara Bio Inc.). The PCR fragment was subcloned and sequenced in pGEM®-T (Promega). The amplification introduced an XbaI site that was used to introduce the *pNFP* fragment in the pCambia2202 binary vector. In the same way, the extracellular part of *NFP* was obtained using NFPfor_EcoRV: 5′-TTGATATCATTTCCTCACAACAATGTC-3′ and NFPrev_EcoRV 5′-AAGATATCAGCACTTCCTAGGCTGATAC-3′. The PCR fragment obtained was subcloned and sequenced in the pGEM®-T vector, digested by EcoRV and subcloned in p2202-pNFP using the SmaI site from the vector polylinker. The kinase part of *NFP* was amplified using the primers NFP3′for 5′-AATTGGTATCAGCCTAGGAAGTGCT-3′ and NFP3′rev 5′-AACCTAGGGGCCACAATAGAGTATG-3′, subcloned and sequenced in pGEM®-T, then introduced in p2202-pNFP-NFP by using AvrII restriction sites.

pNFP:SYM10-NFP and pNFP:LYK3-NFP were obtained in parallel, in a similar way, except that the SYM10 and LYK3 extracellular domains were amplified with the primers Sym10for_EcoRV 5′-TTGATATCAATTTCACAACAATGGCTAT-3′ and NFPrev_EcoRV 5′-AAGATATCAGCACTTCCTAGGCTGATAC-3′ for SYM10 (the two genes are so homologous that the same AvrII restriction site could be used for subcloning the *NFP* kinase part as described above) for *SYM10*, and LYK3for 5′-AAGATATCCAATGAATCTCAAAAATGGATTACT-3′ and LYK3rev 5′-AAGATATCAACCTAGGAATATTCCTGCCATAGCTAT-3′ for *LYK3*.

#### For the Multisite Gateway® technology (Invitrogen)

Promoter sequences were introduced in the pDONR™P4-P1R, the extracellular domain (up to the juxtamembrane domain) in pDONR™221 and the kinase domain in pDONR™P2R-P3. To do so, all fragments were amplified using either TaKaRa LaTaq™ (Takara Bio Inc.) or Phusion™ High Fidelity DNA polymerase (Finnzymes), first subcloned and sequenced in the pGEM®-T (Promega) vector, then used for BP reaction following the manufacturer's instructions.

1.1 kb of the *NFP* promoter was amplified using the primers attB4f_NFP

5′-GGGGACAACTTTGTATAGAAAAGTTGGGCGATCCTTCGTTGTATTCACTTG-3′ and attB1rev_NFP

5′-GGGGACTGCTTTTTTGTACAAACTTGTGTTTCTTATGGCAAATAACAACCAA-3′. The *NFP* extracellular domain was amplified with the primers attB1for_NFP

5′-GGGGACAAGTTTGTACAAAAAAGCAGGCTTATCTCTTTTCTCTTCCCCTCATAAT-3′ and attB2rev_NFP

5′-GGGGACCACTTTGTACAAGAAAGCTGGGTCTCTCTTCATTTTGAGACAATATACGTA-3′.

The kinase and terminator domain of *NFP* was amplified using the primers attB2for_NFP 5′-GGGGACAGCTTTCTTGTACAAAGTGGTTTTGAATAGAAGTACTTCATCGTCC-3′ and attB3rev_NFP

5′-GGGGACAACTTTGTATAATAAAGTTGTTGGCCACAATAGAGTATGGGT-3′.

*SYM10* ECD was obtained by amplification with the primers attB1for_SYM10 5′-GGGGACAAGTTTGTACAAAAAAGCAGGCTTAGCATTTCTTCACAATTTCACAACAATG-3′ and attB2rev_SYM10

5′GGGGACCACTTTGTACAAGAAAGCTGGGTCTCTCTTCATTTTCAGACAATATACA-3′,

SYM10 kinase fragment was obtained by amplification with the primers attB2for_SYM10 5′-GGGGACAGCTTTCTTGTACAAAGTGGTTTTGAATAGGAGTACTTCATTGGCG-3′ and attB3rev_SYM10 5′-GGGGACAACTTTGTATAATAAAGTTGTGTGTTACCTTCACTTGAATGAATATC-3′. The three “ENTRY clones” generated in that way were then used for a Multisite Gateway® LR recombination reaction following the manufacturer's instructions and using pAM-pAT-multi (kindly provided by L. Deslandes (LIPM)) as a destination vector. The recombined sequence obtained was then taken out of pAM-pAT-multi by AscI and PmeI digestion and subcloned in a pCambia2202 vector-modified for its multiple cloning sites to introduce an AscI site- using the AscI and SmaI restrictions sites.

For the LysM swaps:

ENTRY clones with swapped LysM domains were obtained as follows:

-for LysM1 swap: a first round of amplifications of pGEMT-SYM10 by attB1f_SYM10 5′-GGGGACAAGTTTGTACAAAAAAGCAGGCTTAGCATTTCTTCACAATTTCACAACAATG-3′ and LysM1@sens 5′-ACATGAAGGAGGAGAATC-3′, p2201-NFP by LysM1for 5′-GATTCTCCTCCTTCATGT-3′ and LysM2@sens 5′-AGTGCAACCACAAGTTAC-3′, and pGEMT-SYM10 by LysM2for 5′-GTAACTTGTGGTTGCACT-3′ and attB2rev_SYM10

5′GGGGACCACTTTGTACAAGAAAGCTGGGTCTCTCTTCATTTTCAGACAATATACA-3′ was performed using Phusion™ High Fidelity DNA polymerase (Finnzymes), then a mix of 5 µL of each PCR product obtained was used as a template for a new PCR using attB1for_SYM10 and attB2rev_SYM10. The PCR product obtained was subcloned in pGEM®-T vector and sequenced. Verified clones were then used for BP reaction with the pDONR™221 (Invitrogen) vector, following the manufacturer's instructions. The ENTRY clone obtained was then used for Multisite Gateway® LR recombination reaction as described above.

-similarly, for LysM2 swap: first round of amplification with attB1f_SYM10 5′-GGGGACAAGTTTGTACAAAAAAGCAGGCTTAGCATTTCTTCACAATTTCACAACAATG-3′ and LysM2@sens 5′-AGTGCAACCACAAGTTAC-3′ with pGEMT-SYM10, LysM2for 5′-GTAACTTGTGGTTGCACT-3′ and LysM3@sens 5′-CAATTGATTCTTTGAAGGGCA-3′ with p2201-NFP, and LysM3for 5′-TGCCCTTCAAAGAATCAATTG-3′ and attB2rev_SYM10

5′-GGGGACCACTTTGTACAAGAAAGCTGGGTCTCTCTTCATTTTCAGACAATATACA-3′ with pGEMT-SYM10 and second round with attB1for_SYM10 and attB2rev_SYM10 (and a mix of 5 µL of each PCR product as a template)

-for LysM3 swap: attB1for_SYM10 and LysM3@sens 5′-CAATTGATTCTTTGAAGGGCA-3′ with pGEMT-SYM10, LysM3for 5′-TGCCCTTCAAAGAATCAATTG-3′ and LysM3ext@sens 5′-TCCATTTGAAGATGGTTG-3′ and p2201-NFP, and LysM3extfor 5′-CAACCATCTTCAAATGGA-3′ and attB2rev_SYM10 with pGEMT-SYM10 as a first round of amplification and attB1f_SYM10 and attB2rev_SYM10 as a second round on a mix of 5 µL of each previous PCR products as a template.

Site directed mutagenesis was performed:

on the pENTRY 221-SYM10 clone using the primers:

-For E141K sym10mutE 5′-TATGTGGAAATGAAAAATTTCAACCC-3′ and sym10mutE@sens 5′-GGGTTGAAATTTTTCATTTCCACATA-3′

- for I156T: sym10mutI 5′-AAATCTATTGCCACCAGAAACCAAAGTTGTTGT-3′ and sym10 mutI@sens 5′-ACAACAACTTTGGTTTCTGGTGGCAATAGATTT-3′

- for P154L: sym10mutP 5′-AAATCTATTGCCACTAGAAATCAAAGTTGTTGT-3′ and sym10mutP@sens 5′-ACAACAACTTTGATTTCTAGTGGCAATAGATTT-3′

on the pENTRY 221-NFP clone using the primers:

-For K141E NFP_mutKfor 5′-ACCAATTATCTTGAATTTGAAAATTTCAACCCC-3′ and NFP_mutK rev 5′-GGGGTTGAAATTTTCAAATTCAAGATAATTGGT-3

-for T156I: NFP_mutTfor 5′-TATTGCCACTAGACATCAAAGTTTCAGTC-3′ for and NFP_mutTrev 5′-GACTGAAACTTTGATGTCTAGTGGCAATA-3′ rev

for L154P: NFP_mutLfor 5′-TATTGCCACCAGACACCAAAGTTTCAGTC-3′ and NFP_mutLrev 5′-GACTGAAACTTTGGTGTCTGGTGGCAATA-3′

using the Pfu Turbo DNA polymerase (Tm = 50°C, 20 cycles) and the QuickChange® Site Directed Mutagenesis kit from Stratagene, following the manufacturer's instructions. The mutated versions were then used for Multisite Gateway® LR recombination reaction as described above.

### *nfp lyk3* genotyping

Genotyping of the *lyk3-1* mutation [25] was performed using dCAPS analysis (with the help of dCaps Finder 2.0 software (http://helix.wustl.edu/dcaps/dcaps.html). “Rapidly extracted” genomic DNA (adapted from [43]) was amplified using the primers dcaps_lyk3_for 5′-GCATGCTACTGGTAGTGCTG-3′ and dcaps_lyk3_rev

5′-CTCTAAGTTCTGCATAATAGAAAGCT-3′ (Tm = 50°C, 35 amplification cycles, Promega GoTaq Enzyme). 10 µL of the PCR product obtained was then digested by HindIII, which cleaves only when the *lyk3-1* mutation is present (creating a shift from 158 bp to 134 bp). Digestion fragments were resolved on a 3.4% agarose gel.

*nfp-1* genotyping was performed as described in [11].

### NF treatment

Purified Nod factors from *Rhizobium leguminosarum* bv. *viciae*, *S. meliloti nodH* and *S. meliloti* wild type rhizobia were kindly provided by F. Maillet (LIPM), as 10^−3^ M stock solutions. 1 µL of the stock solution was used to obtain a 10^−8^ M or 10^−9^ M working solution in sterile water.

Transformed roots were incubated in small pots with 20 ml of this solution for 16 h (overnight) in a growth chamber at 25°C with 16 h light/8 h dark cycle.

### Statistical analysis

Chi-square test of independence was used for analysis of qualitative results (such as GUS positive plants). For comparison of means (nodulation tests), non parametric tests were used for non normal distribution: a Mann-Whitney test in the case of the comparison of two independent samples and a Kruskal-Wallis test for the comparison of multiple samples (such as different point mutations). Analysis of variance (ANOVA) was used for the comparison of multiple samples with a normal distribution. Mann-Whitney, Kruskal-Wallis and ANOVA tests were performed using the Statgraphics Centurion software (Sigma Plus).

### Microscopy methods

Histochemical tests were performed as described before [28].

Whole root segments were observed after histochemical staining for β-glucuronidase (GUS) activity for 3 hours at 37°C with the substrate magenta-glucA (5 bromo-4 chloro-3 indolyl glucuronide, cyclohexylammonium salt) (Duchefa Biochemie, The Netherlands). To visualise bacterial infection, whole roots were lightly fixed under vacuum for 15′ with 1.5% glutaraldehyde phosphate buffered solution followed by one hour fixation. Histochemical staining of β-galactosidase activity expressed by the plasmid PXLGD4 was performed as described in [19].

Whole root segments were observed with a Leica MZFLIII stereomicroscope (Leica Microsystems, Wetzlar, Germany).

For further analysis, 5 mm fragments were transversally and longitudinally sectioned. 70 µm thick and 5 µm thick sections were made using respectively a vibratome (Leica VT1000S) or a microcut (2040 Reichert Jung). The fragments were embedded in 4% agarose solution for the 70 µm thick sections and in Technovit 7100 resin (Heraeus Kulzer, Wehrheim, Germany) for the 5 µm sections. Observations were performed on a Zeiss Axioplan2 imaging microscope.

### Accession numbers

Sequence data used for this article can be found in the GenBank/EMBL data libraries under the following accession numbers: *Pisum sativum SYM10* gene, cultivar Frisson: AJ575251; *Medicago truncatula LYK3* (complete cds): AY372406; *Medicago truncatula NFP*: DQ496250.

## Supporting Information

Figure S1 ***pMtENOD11:GUS*** **induction is similar between wild type and pNFP:SYM10-NFP** ***nfp*** **plants following Nod factor treatment.** *nfp pMtENOD11:GUS* non transformed plants (A, D, G), wild type *pMtENOD11:GUS* plants (B, E, H) and *nfp pMtENOD11:GUS* plants transformed with the pNFP:SYM10-NFP construct (C, F, I). Roots were stained for GUS activity (blue) 16 h post treatment with 10^−9^ M purified Nod factors from wild type *S. meliloti* (A, B, C), *S. meliloti nodH* (D, E, F) or 10^−8^ M purified Nod factors from *Rhizobium leguminosarum* bv. *viciae* (G, H, I). Bars = 500 µm.
(PDF)

Figure S2 **Comparison of NFP and SYM10 extracellular domains, and LysM2 with** ***Medicago*** **or** ***Vicia*** **spp. sequences.** (A) Amino acid alignment of NFP (*M. truncatula*) and SYM10 (pea) whole extracellular domains showing the consensus sequence. LysM domains are boxed. The lysine (K) to glutamic acid (E) and the leucine (L) to proline (P) variations are shown by arrows. TM = transmembrane domain. (B) Amino acid alignment of LysM2 domains of different NFP homologs from *Medicago* spp. and *Vicia* spp. The amino acids with conserved biochemical properties (corresponding to Lys141, Leu154 and Thr156 in (A)) in *Medicago* spp. are boxed and shown by arrows. Amino acid sequence alignments were made with the Multalin software (Corpet, 1988). Corpet, F. (1988). Multiple sequence alignment with hierarchical clustering. Nucleic Acids Res 16, 10881–10890.
(PDF)

Figure S3 **Complementation for** ***pMtENOD11:GUS*** **and nodulation of** ***nfp*** **plants by different chimeric constructs at 7 dpi.** *nfp pMtENOD11:GUS* plants were transformed with an empty vector (A), pNFP:NFP-NFP (B), pNFP:SYM10-I156T-NFP (C), pNFP:SYM10-P154L-NFP (D), pNFP:SYM10-E141K-NFP (E), pNFP:NFP-T156I-NFP (F), pNFP:NFP-L154P-NFP (G) and tested for *pMtENOD11:GUS* activity (in magenta) and nodule formation at 7 dpi. Bar = 1 mm.
(PDF)

Figure S4 **Complementation for** ***pMtENOD11:GUS*** **and nodulation of** ***nfp*** **plants by different chimeric constructs at 21 dpi.** *nfp pMtENOD11:GUS* plants were transformed with pNFP:SYM10-NFP (A), pNFP:SYM10-P154L-NFP (B), pNFP:SYM10-E141K-NFP (C), pNFP:SYM10-I156T-NFP (D), pNFP:NFP-T156I-NFP (E), pNFP:NFP-L154P-NFP (F) and tested for *pMtENOD11:GUS* activity (in magenta) and nodule formation at 21 dpi. B, C and E are 70 µm-thick sections. Bar = 100 µm.
(PDF)

Table S1 **Frequency of** ***pMtENOD11:GUS*** **expression in** ***nfp pMtENOD11:GUS*** **plants transformed with different versions of the pNFP:NFP-NFP or pNFP:SYM10-NFP constructs, in response to wild type** ***S. meliloti*** **(7 dpi).**
(DOC)
