# Supplementary material for: Contribution of NFP LysM Domains to the Recognition of Nod Factors during the Medicago truncatula/Sinorhizobium meliloti Symbiosis
Source: PLoS One. 2011 Nov 8;6(11):e26114. doi: 10.1371/journal.pone.0026114 (PMC3210742; doi:10.1371/journal.pone.0026114)
Supplement: Figure S1 — pMtENOD11:GUS induction is similar between wild type and pNFP:SYM10-NFP nfp plants following Nod factor treatment. nfp pMtENOD11:GUS non transformed plants (A, D, G), wild type pMtENOD11:GUS plants (B, E, H) and nfp pMtENOD11:GUS plants transformed with the pNFP:SYM10-NFP construct (C, F, I). Roots were stained for GUS activity (blue) 16 h post treatment with 10−9 M purified Nod factors from wild type S. meliloti (A, B, C), S. meliloti nodH (D, E, F) or 10−8 M purified Nod factors from Rhizobium leguminosarum bv. viciae (G, H, I). Bars = 500 µm. (PDF) [file pone.0026114.s001.pdf]

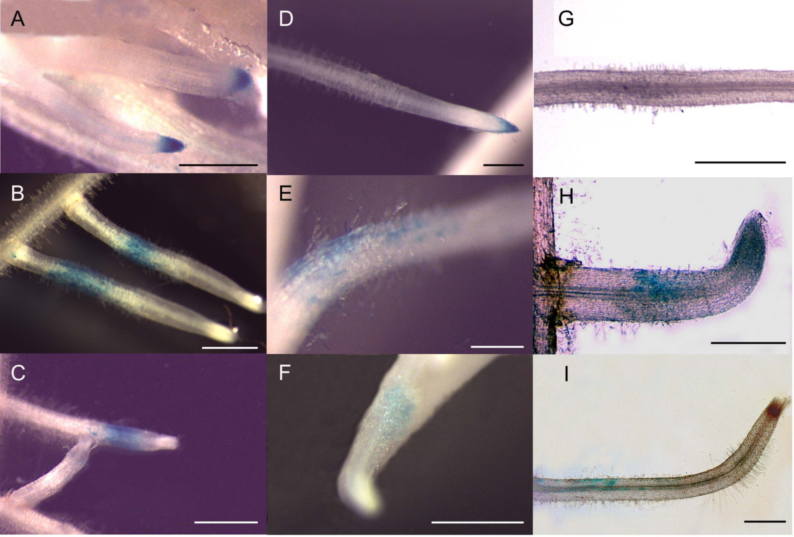

Figure S1. *pMtENOD11:GUS* induction is similar between wild type and pNFP:SYM10-NFP *nfp* plants following Nod Factor treatment.

nfp pMtENOD11:GUS non transformed plants (A, D, G), wild type *pMtENOD11:GUS* plants (B, E, H) and *nfp pMtENOD11:GUS* plants transformed with the pNFP:SYM10-NFP construct (C, F, I). Roots were stained for GUS activity (blue) 16 h post treatment with 10^{-9} M purified Nod factors from wild type *S. meliloti* (A, B, C), *S. meliloti nodH* (D, E, F) or 10^{-8} M purified Nod factors from *Rhizobium leguminosarum* bv. *viciae* (G, H, I). Bars = 500µm.
