# Supplementary material for: Contribution of NFP LysM Domains to the Recognition of Nod Factors during the Medicago truncatula/Sinorhizobium meliloti Symbiosis
Source: PLoS One. 2011 Nov 8;6(11):e26114. doi: 10.1371/journal.pone.0026114 (PMC3210742; doi:10.1371/journal.pone.0026114)
Supplement: Figure S2 — Comparison of NFP and SYM10 extracellular domains, and LysM2 with Medicago or Vicia spp. sequences. (A) Amino acid alignment of NFP (M. truncatula) and SYM10 (pea) whole extracellular domains showing the consensus sequence. LysM domains are boxed. The lysine (K) to glutamic acid (E) and the leucine (L) to proline (P) variations are shown by arrows. TM = transmembrane domain. (B) Amino acid alignment of LysM2 domains of different NFP homologs from Medicago spp. and Vicia spp. The amino acids with conserved biochemical properties (corresponding to Lys141, Leu154 and Thr156 in (A)) in Medicago spp. are boxed and shown by arrows. Amino acid sequence alignments were made with the Multalin software (Corpet, 1988). Corpet, F. (1988). Multiple sequence alignment with hierarchical clustering. Nucleic Acids Res 16, 10881–10890. (PDF) [file pone.0026114.s002.pdf]

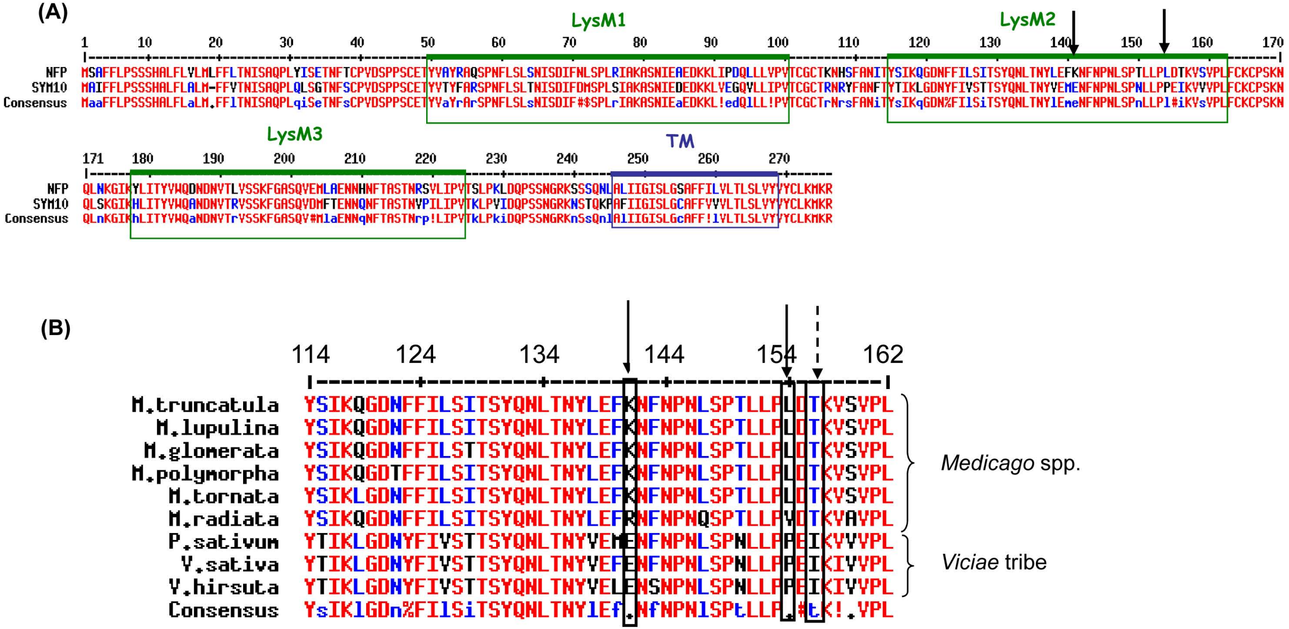

Figure S2. Comparison of NFP and SYM10 extracellular domains, and LysM2 with *Medicago* or *Vicia* spp. sequences.

(A) Amino acid alignment of NFP (*M. truncatula*) and SYM10 (pea) whole extracellular domains showing the consensus sequence. LysM domains are boxed.

The lysine (K) to glutamic acid (E) and the leucine (L) to proline (P) variations are shown by arrows. TM = transmembrane domain.

(B) Amino acid alignment of LysM2 domains of different NFP homologs from *Medicago* spp. and *Vicia* spp.. The amino acids with conserved biochemical properties (corresponding to Lys141, Leu154 and Thr156 in (A)) in *Medicago* spp. are boxed and shown by arrows.

Amino acid sequence alignments were made with the Multalin software (Corpet, 1988).

Corpet, F. (1988). Multiple sequence alignment with hierarchical clustering. Nucleic Acids Res 16, 10881-10890.
