# Supplementary material for: Contribution of NFP LysM Domains to the Recognition of Nod Factors during the Medicago truncatula/Sinorhizobium meliloti Symbiosis
Source: PLoS One. 2011 Nov 8;6(11):e26114. doi: 10.1371/journal.pone.0026114 (PMC3210742; doi:10.1371/journal.pone.0026114)
Supplement: Figure S4 — Complementation for pMtENOD11:GUS and nodulation of nfp plants by different chimeric constructs at 21 dpi. nfp pMtENOD11:GUS plants were transformed with pNFP:SYM10-NFP (A), pNFP:SYM10-P154L-NFP (B), pNFP:SYM10-E141K-NFP (C), pNFP:SYM10-I156T-NFP (D), pNFP:NFP-T156I-NFP (E), pNFP:NFP-L154P-NFP (F) and tested for pMtENOD11:GUS activity (in magenta) and nodule formation at 21 dpi. B, C and E are 70 µm-thick sections. Bar = 100 µm. (PDF) [file pone.0026114.s004.pdf]

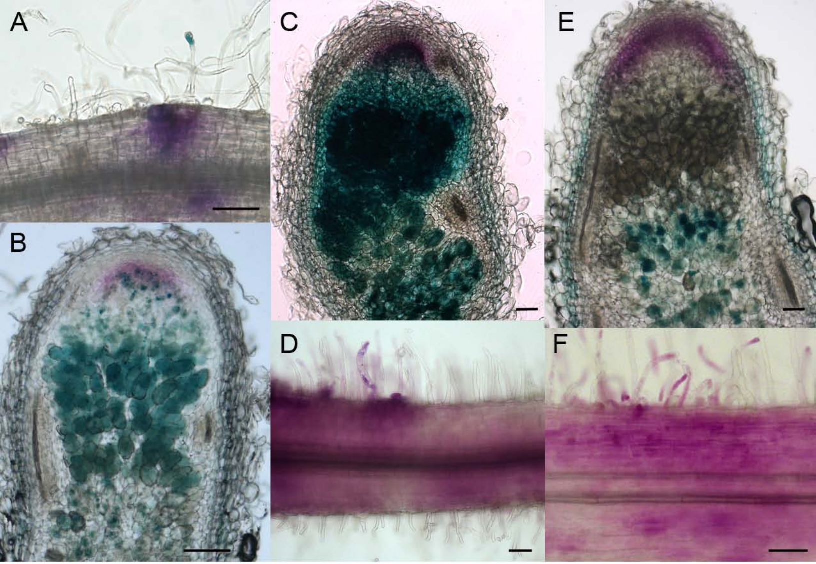

Figure S4. Complementation for *pMtENOD11:GUS* and nodulation of *nfp* plants by different chimeric constructs at 21dpi.

nfp pMtENOD11:GUS plants were transformed with pNFP:SYM10-NFP (A), pNFP:SYM10-P154L-NFP (B), pNFP:SYM10-E141K-NFP (C), pNFP:SYM10-I156T-NFP (D), pNFP:NFP-T156I-NFP (E), pNFP:NFP-L154P-NFP (F) and tested for *pMtENOD11:GUS* activity (in magenta) and nodule formation at 21 dpi.

B, C and E are 70 µm-thick sections. Bar = 100 µm.
