# Supplementary material for: Contribution of NFP LysM Domains to the Recognition of Nod Factors during the Medicago truncatula/Sinorhizobium meliloti Symbiosis
Source: PLoS One. 2011 Nov 8;6(11):e26114. doi: 10.1371/journal.pone.0026114 (PMC3210742; doi:10.1371/journal.pone.0026114)
Supplement: Table S1 — Frequency of pMtENOD11:GUS expression in nfp pMtENOD11:GUS plants transformed with different versions of the pNFP:NFP-NFP or pNFP:SYM10-NFP constructs, in response to wild type S. meliloti (7 dpi). (DOC) [file pone.0026114.s005.doc]

**Table S1. Frequency of *pMtENOD11:GUS*  expression in *nfp pMtENOD11:GUS* plants transformed with different versions of the pNFP:NFP-NFP or pNFP:SYM10-NFP constructs, in response to wild type *S. meliloti*** (7 dpi).

|  | **pNFP:NFP-NFP** | | **pNFP:SYM10-NFP** | |
| --- | --- | --- | --- | --- |
|  | Classical cloning | Gateway® cloning | Classical cloning | Gateway® cloning |
| GUS positive plants | 30/38 (79%) | 23/33 (70%) | 33/43 (77%) | 17/22 (77%) |

Chi-square test of independence gives no significant difference in the proportions of the Gateway® or classical cloning techniques, neither for pNFP:NFP-NFP and pNFP:SYM10-NFP transformed plants.

The total number of plants tested comes from 3 to 9 independent experiments.
